# Spatial Distribution Pattern Evolution of the Population and Economy in Russia since the 21st Century

**DOI:** 10.3390/ijerph20010684

**Published:** 2022-12-30

**Authors:** Nanchen Chu, Xiangli Wu, Pingyu Zhang, Shuang Xu, Xiaonan Shi, Bo Jiang

**Affiliations:** 1 College of Geographical Sciences, Harbin Normal University, Harbin 150025, China; 2 Northeast Institute of Geography and Agroecology, Chinese Academy Sciences, Changchun 130102, China; 3 College of Resources and Environment, University of Chinese Academy of Sciences, Beijing 100049, China; 4 School of Public Administration and Law, Northeast Agricultural University, Harbin 150030, China

**Keywords:** population distribution, economic development, spatial pattern, Russia

## Abstract

Under the background of “the Belt and Road” and “China, Mongolia and Russia economic corridor” initiatives, this paper studied the spatial distribution pattern evolution of population and economy in Russia since the 21st century, which could provide implications for the regional development planning, economic optimization layout, energy resource development, transportation infrastructure construction between China and Russia. Combined with the panel data of population, GDP, land area, etc., we used the gravity center analysis, geographic concentration degree, and inconsistency index to study Russia’s population pattern evolution trend, economic pattern evolution trend, spatial inconsistency types of population distribution and economic development. The results and conclusions are as follows. Russia’s population and economic gravity centers have migrated towards the northwest direction. Russia’s population and economic distribution pattern presents the unbalanced development trend, which could be characterized by the differentiation pattern of “High West, Low East” and “High South, Low North” divided by the Ural Federal District. In the southwest areas of Russia, the population concentration degree is higher than the economic concentration degree in most federal subjects. In the northeast areas of Russia, the economic concentration degree is higher than the population concentration degree in most federal subjects.

## 1. Introduction

Population distribution and economic development are important contents in studying the regional differentiation pattern [1]. Population distribution and economic development show the relationship of mutual benefit, mutual coexistence and mutual causation by interacting with each other and restricting each other. Population agglomeration could provide the labor resources for economic development, and it is one of the important driving mechanisms in the economic development. Economic development could contribute to employment and income increasing, and it could also improve the social environment [2,3]. The regional spatial pattern evolution is closely related to the spatial elements such as population density and economic density [4]. Studying the population spatial pattern, economic spatial pattern, and their inconsistency spatial pattern could provide an important reference for understanding the regional spatial pattern evolution trend. They could also provide an important basis for formulating reasonable scientific policies for regional population distribution and regional economic development.

The issues of population distribution and economic development are important topics in geography, demography, economics and other subjects. Neoclassical economic theory [5], Push-Pull theory [6], and Lee’s migration law [7] are the most important theories to study population distribution and economic development. The earliest research on the relationship between population distribution and economic development came from Adam Smith in the second half of the 18th century. Adam Smith analyzed the relationship between the regional economic development, regional population growth, and regional labor growth [8]. Adam Smith believed that population increase could promote economic growth, and it was one of the most important symbols of economic prosperity [8]. But Malthus proposed that excessive population growth could inhibit economic development [9]. Since then, from diversified research perspectives, scholars studied the relationship between population growth and economic growth [10,11], the relationship between population shrinking and economic development [12,13], and the impact of population aging on the economic growth [14]. These studies have shown the dynamic, diversified and small-scale trend from the qualitative analysis of the population spatial structure and economic spatial structure, to the quantitative discussions of their internal correlation mechanisms, rules and factors [15]. The research systems of population distribution and economic development have been constantly enriching and improving.

To sum up, the studying perspectives of population distribution and economic development concentrate on the relationship between population growth and economic growth, the relationship between population shrinking and economic development, and the impact of population aging on economic growth, etc. These studies have shown the dynamic, diversified, large-medium-small scale development trends, which are from the qualitative analysis to the quantitative discussions. However, compared with economics subject, demography subject, trade subject, management subject and other subjects, geography subject has relatively few studies on population distribution and economic development. In addition, the existing geographical research of population distribution and economic development are mainly conducted in economically developed countries, such as Europe and the United States. The literature has relatively few studies focusing on the population distribution and economic development of Russia. The literature on population distribution and economic development of Russia mainly focuses on one aspect of population distribution or economic development. In this context, we comprehensively studied Russia’s population pattern evolution trend, economic pattern evolution trend, spatial inconsistency types of population distribution and economic development.

Russia is an important neighbor country in the north of China. It has the largest land area and the most abundant resources in the world. However, due to the factors such as natural resource endowments, historical social conditions, military strength, and international environment, Russia’s population development pattern and economic development pattern both show great spatial differences in the east-west direction and south-north direction. After the collapse of the Soviet Union, Russia experienced a population crisis and economic crisis under the background of “shock therapy” [16]. At the beginning of the 21st century, relying on the export of resources and energy, economic development showed a rapid growth trend in Russia. However, in recent years, the economic phenomenon of “heavy industry was too heavy, the light industry was too light, and agriculture was lagging behind for a long time” occurred in Russia’s economic development [17,18]. Russia also suffered the economic development disadvantages of “economic raw materials, export raw materials, investment raw materials” [17,18]. At the same time, Russia’s population changed from a growth trend lasting more than 100 years in the Soviet Union to a downward trend in the past 20 years (1991–2009). Russia’s population structure was increasingly aging under the background of declining average life expectancy. Russia’s population gender ratio was unbalanced, and its life expectancy ratio was also unbalanced [19]. Spatially, the densely populated hot areas were distributed along the Volga River and in the south of the Ural Federal District. The sparsely populated cold areas were distributed in the west of the Siberian Federal District, South Federal District and North-Caucasian Federal District [20]. In addition, Siberia Federal District and Far East Federal District suffered from a population crisis, due to the factors of population back-flow to Europe and population restrictions from foreign migrant labor [21,22].

In this paper, based on the panel data of Russia’s population, Russia’s GDP, Russia’s land area, etc. from 2002 to 2020, Russia’s population pattern evolution trend, Russia’s economic pattern evolution trend, Russia’s spatial inconsistency types of population distribution and economic development, were studied by using the gravity center analysis, geographic concentration degree, inconsistency index, etc. The paper first used the gravity center method to simulate the spatial dynamic pattern of Russia’s population gravity center and Russia’s economic gravity center. Then it calculated the population concentration degree and economic concentration degree in Russian 83 federal subjects. Next, combined with the inconsistency index of the population concentration degree and economic concentration degree, we classified the population-economic inconsistency types in Russian 83 federal subjects. Finally, we suggested policies and strategies that can boost the population and economic coordinated development in Russia. In the theory, this research could enrich the understanding of Russia’s population distribution pattern and Russia’s economic development pattern. It also could provide some basic contents to study the population and economic relations of the other countries in the world. In the practice, under the background of “the Belt and Road” and “China, Mongolia and Russia economic corridor” initiatives, clarifying Russia’s population distribution pattern evolution trend and Russia’s economic development pattern evolution trend could provide important support for exploring the long-term, stable and multi-field cooperation paths between China and Russia. This research could be helpful to seek important breakthrough points for the bilateral economic trade cooperation between China and Russia. This research could provide scientific reference for constructing the important cross-border economic cooperation nodes, economic cooperation lines and economic cooperation zones between China and Russia. It also could provide policy implications for regional development planning, economic optimization layout, energy resource development, transportation infrastructure construction, and science technology cooperation in the neighboring regions between China and Russia.

## 2. Materials and Methods

### 2.1. Study Area

**Figure 1:**
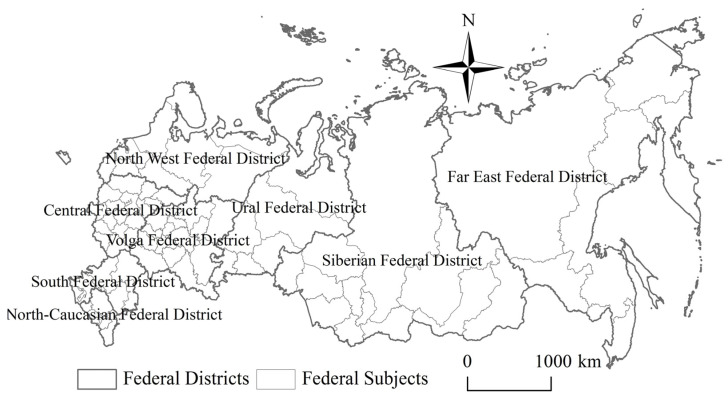
Sketch map of the study area.

Russia has eight federal districts, including the Central Federal District, Ural Federal District, Volga Federal District, North West Federal District, South Federal District, North-Caucasian Federal District, Siberian Federal District, and Far East Federal District. It has eighty-five federal subjects, including forty-six regions, three municipality cities, one autonomous oblast, nine territories, twenty-two republics, and four autonomous areas (Due to the fact that the Sevastopol city and the Republic of Crimea are in dispute with the Ukrainian State, Sevastopol city and the Republic of Crimea are not selected as the objects in this study) (Figure 1). In 2020, Russia had a land area of 17.13 million km^2^, a population of 146 million, employees of 71 million, a population density of 8.5 persons/km^2^, a gross regional product of 94.83 trillion rubles, a fixed capital investment of 20.12 trillion rubles, the retail trade turnover of 33.87 trillion rubles, per capita monthly monetary income of 35,676 rubles, and per capita monthly monetary expenditure of 27,154 rubles.

### 2.2. Research Methods

#### 2.2.1. Gravity Center Method

The gravity center method is derived from the gravity center concept in physics subject. The forces around the regional spatial gravity center are relatively balanced in all directions. The gravity center method has significant advantages in studying the spatial elements evolution trend. It can accurately judge the spatial distribution law and spatial evolution trend of elements by measuring the distance and direction of the elements’ gravity center deviating from the geometric gravity center. It has become an important analysis tool to discuss the spatial elements evolution in the process of regional development [23]. As an evaluation index to measure the spatial elements distribution, the temporal change of the regional gravity center could reflect the spatial evolution track of regional development [24]. The gravity center method is used to measure the overall distribution pattern evolution trend of certain elements in the region. The population gravity center and economic gravity center refer to the stress point where the population level and economic level could maintain spatial balance [25]. As for Russia, the population gravity center is the resultant force point of the population sub vector within every federal subject [26]. The economic gravity center is the resultant force point of the economic sub vector within every federal subject [26]. Based on the population data and GDP data of eighty-three federal subjects, we measured Russia’s population gravity center and Russia’s economic gravity center since the 21st century. First, we took the geographical coordinates of administrative centers of eighty-three federal subjects as the population focus and economic focus of every federal subjects. Then we studied the spatial dynamic evolution process of Russia’s population gravity center, Russia’s economic gravity center, and their deviation degree from Russia’s geographical gravity center. The formula is as follows:

(1) $$X=\frac{\sum G_{i}\times x_{i}}{\sum G_{i}},\;Y=\frac{\sum G_{i}\times y_{i}}{\sum G_{i}}$$

where *x_i_* is the longitude of *i* federal subject. *y_i_* is the latitude of *i* federal subject. *G_i_* is some certain elements values (such as population, or GDP) of *i* federal subject. *X* is the longitude of the gravity center of some certain elements values (such as population, or GDP). *Y* is the latitude of the gravity center of some certain elements values (such as population, or GDP).

#### 2.2.2. Geographical Concentration Degree

Geographic concentration degree is one of the most important methods to measure regional population spatial distribution pattern and regional economic spatial distribution pattern. Geographic concentration degree, including population concentration degree and economic concentration degree, is the important method that comprehensively considers the population, economic total and regional area. It is also an effective indicator to measure the population spatial distribution and economic spatial distribution [27]. The population concentration degree is a method to measure the regional concentration degree of population elements. Considering factors such as regional population and regional area, it could not only reflect the spatial distribution characteristics of the population, but also reflect the role and status of small units at the same level in the region [28]. The economic concentration degree is a method to measure the regional concentration degree of economic elements. Considering factors such as regional GDP and regional area, it could not only reflect the spatial distribution characteristics of the economy, but also reflect the role and status of small units at the same level in the region [29]. As for Russia, comprehensively considering the population, economic total, regional area and other factors of every federal subject, we used the population concentration degree to measure the population distribution pattern evolution trend in Russia’s geographical space. We used the economic concentration degree to measure the economic distribution pattern evolution trend in Russia’s geographical space. According to the results of the population concentration degree and economic concentration degree, we could explain the population status and economic roles of different federal subjects. The formula is as follows:

(2) $$C_{POPi}=\frac{POP_{i}/\sum POP_{i}}{S_{i}/\sum S_{i}},\;C_{GDPi}=\frac{GDP_{i}/\sum GDP_{i}}{S_{i}/\sum S_{i}}$$

where *C_POPi_* is the population concentration degree of *i* federal subject. *C_GDPi_* is the economic concentration degree of *i* federal subject. The greater the *C_POPi_*, the greater the relative population density of *i* federal subject, and the greater the population concentration trend of *i* federal subject. The greater the *C_GDPi_*, the greater the relative economic density of *i* federal subject, and the greater the economic concentration trend of *i* federal subject. *POP_i_* is the population number of *i* federal subject. *GDP_i_* is the *GDP* value of *i* federal subject. *S_i_* is the land area of *i* federal subject.

#### 2.2.3. Inconsistency Index

The inconsistency index mainly reflects the difference and imbalance of urban population agglomeration level and economic agglomeration level, and it further reflects the difference between urban population and economic development level [30]. The inconsistency index is used to study the coupling relationship of the population spatial pattern and economic spatial pattern, that is, the overall centralization and decentralization of population elements and economic elements in the regional space [31]. The inconsistency index is defined as the ratio of population concentration degree and economic concentration degree to measure the inconsistency status between population elements and economic elements [32]. As for Russia, first, we calculated the proportion of population concentration degree and economic concentration degree as the inconsistency index of every federal subject. Then we used the population-economy inconsistency index to explain the consistency degree or inconsistency degree of the population spatial distribution and the economic spatial distribution in every federal subject. Finally, through the population-economy inconsistency index of every federal subject, we studied the population and economic agglomeration differences and disequilibrium state in every federal subject. The formula is as follows:

(3) $$I_{i}=\frac{C_{POPi}}{C_{GDPi}}$$

where *I_i_* is population-economy inconsistency index of *i* federal subject. The greater the deviation degree of *I_i_* from 1, the greater the inconsistency degree of population and economic distribution of *i* federal subject. The greater the close degree of *I_i_* to 1, the greater the consistency degree of population and economic distribution of *i* federal subject. The population-economy inconsistency index of *i* federal subject could be further subdivided into five classes: population agglomeration is far lower than economic agglomeration (*I_i_* is lower than 0.5), population agglomeration is slightly lower than economic agglomeration (*I_i_* is between 0.5 and 0.8), population agglomeration and economic agglomeration are basically the same (*I_i_* is between 0.8 and 1.2), population agglomeration is slightly higher than economic agglomeration (*I_i_* is between 1.2 and 2), population agglomeration is far higher than economic agglomeration (*I_i_* is greater than 2) [33]. *C_POPi_* and *C_GDPi_* ditto.

### 2.3. Data Sources

The data of population, GDP, land area, longitude, latitude, and other indicators of Russia, eight federal districts and eighty-three federal subjects all came from the official yearbook “RUSSIA IN FIGURES STATISTICAL HANDBOOK” issued from 2003 to 2021 in the official website of the Russian Federal Bureau of Statistics (Rosstat) “https://www.gks.ru/” (accessed on 1 March 2022).

## 3. Results

### 3.1. Spatial Pattern of Population Gravity Center and Economic Gravity Center

**Figure 2:**
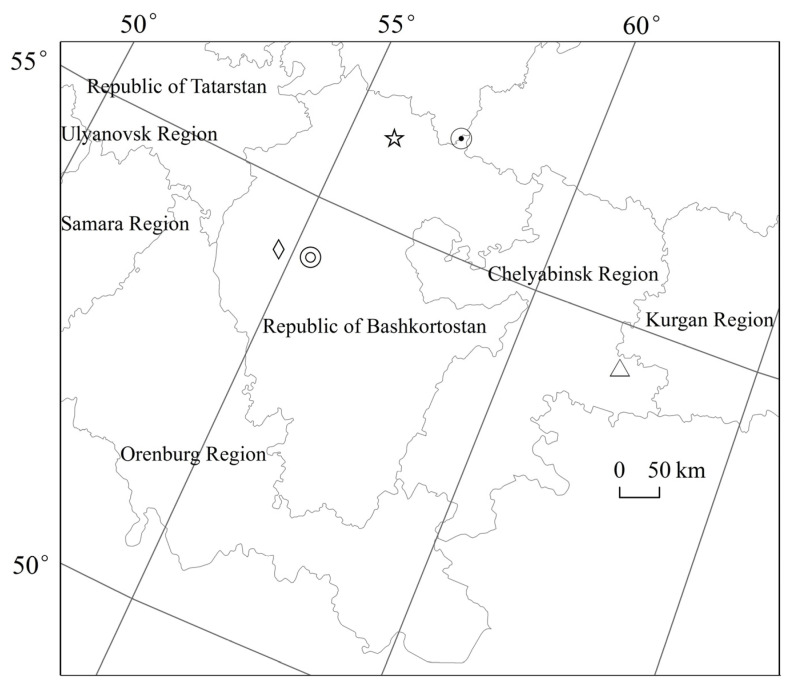
Spatial pattern of Russia’s geographical gravity center, population gravity center, and economic gravity center. Note: △ is Russia’s geographical gravity center. ⊙ is Russia’s economic gravity center in 2002. ☆ is Russia’s economic gravity center in 2020.◎ is Russia’s population gravity center in 2002. ◇ is Russia’s population gravity center in 2020.

Russia’s geographical gravity center is 62°14′34″ E, 54°31′42″ N, which is located in Chelyabinsk Region in the Ural Federal District. From 2002 to 2020, Russia’s population gravity center has been migrating among 54°37′00″ E~55°22′53″ E, 54°15′43″ N~54°20′33″ N. Russia’s economic gravity center has been migrating among 55°15′40″ E~57°42′44″ E, 55°50′38″ N~56°22′47″ N. Russia’s population gravity center and Russia’s economic gravity center are both located in the northwest direction of Russia’s geographical gravity center. Russia’s population gravity center and Russia’s economic gravity center have been gradually migrating away from Russia’s geographical gravity center. The distance between Russia’s population gravity center and Russia’s geographical gravity center has been increasing from 444 km in 2002 to 494 km in 2020. The distance between Russia’s economic gravity center and Russia’s geographical gravity center has been increasing from 368 km in 2002 to 402 km in 2020. Russia’s population gravity center and Russia’s economic gravity center have been migrating towards the northwest direction. From 2002 to 2020, the longitude of Russia’s population gravity center has been migrating 45′, the latitude of Russia’s population gravity center has been migrating 5′, and the distance of Russia’s population gravity center has been migrating 50 km. The longitude of Russia’s economic gravity center has been migrating 46′, the latitude of Russia’s economic gravity center has been migrating 10′, and the distance of Russia’s economic gravity center has been migrating 51 km (Figure 2). In general, the spatial migrating degree, spatial migrating scope, and spatial migrating distance of Russia’s economic gravity center are larger than those of Russia’s population gravity center. Russia’s economic imbalance degree is greater than Russia’s population imbalance degree. Russia’s economic expansion process is relatively rapid, and Russia’s population flow process is relatively slow. However, it should be noted that compared with the vast land area of 17.13 million km^2^ in Russia, the spatial migrating distance of Russia’s population gravity center and economic gravity center could be neglected since the 21st century.

Russia’s population gravity center and Russia’s economic gravity center are migrating towards the northwest direction, which is closely related to Russia’s geographical environment, resource endowment, development intensity, policy system, productivity layout, etc. In the 1990s, the Soviet Union disintegrated into fifteen countries. Some Russian federal subjects which were located in western European areas of Russia became the border federal subjects. The immigrants from other Commonwealth of the Independent States (CIS) poured into the Russian Central Federal District, Russian Volga Federal District, and Russian South Federal District. In these Russian federal districts, the local government increased the investment scale, rebuilt national defense, and reorganized military forces. Later, in order to resist the population crisis and economic crisis affected by the “shock therapy” policy, the Russian government issued “the Main Principles of the Regional Policy of the Russian Federation”. Leningrad Region, Novgorod Region, Kaliningrad Region, etc. established the economic free zones. The Republic of Kalmykia, Republic of Ingushetia, etc. set up the tax-free zones. Russia had gradually transformed from a planned economy country into a market economy country. At the same time, the Russian government stopped subsidizing the northern areas, and the population of the northern areas flowed into the southern areas and western areas quickly. The population of northern European areas of Russia mainly flowed into the St. Petersburg, Leningrad Region and the Central Federal District. The population of the northern Siberian Federal District flowed into the Novosibirsk Region and Ural Federal District. The population of the Far East Federal District flowed into Siberian Federal District, Ural Federal District, and western European federal districts. After 2000, Russia approved “the Concept of Russian Federation Border Cooperation”. Russian government allocated funds to the South Federal District, North-Caucasian Federal District, and other politically sensitive areas. At the same time, the Russian government established the principles of giving priority to industrial development in some federal subjects. For example, the pharmaceutical industry clusters were built in the St. Petersburg and Sverdlovsk Region. The automobile manufacturing industry clusters were built in the Samara Region and Kaluga Region. The household appliance production clusters were built in the Lipetsk Region. The aviation industry clusters were built in the Ulyanovsk Region. The linen industry clusters were built in the Volgograd Region. The internet technology clusters were built in the Republic of Tatarstan. The comprehensive clusters of oil gas, chemistry, electronics, and aviation were built in the Voronezh Region. Moscow Region and Leningrad Region built the Green City Special Economic Zone and St. Petersburg Special Economic Zone. These special economic zones had high-wage employment opportunities, complete medical, science, education and other public facilities, attracting the population, economic factors, etc. to continue to migrate from eastern Asian areas of Russia to central western European areas of Russia.

### 3.2. Spatial Pattern of Population Concentration Degree and Economic Concentration Degree

**Figure 3:**
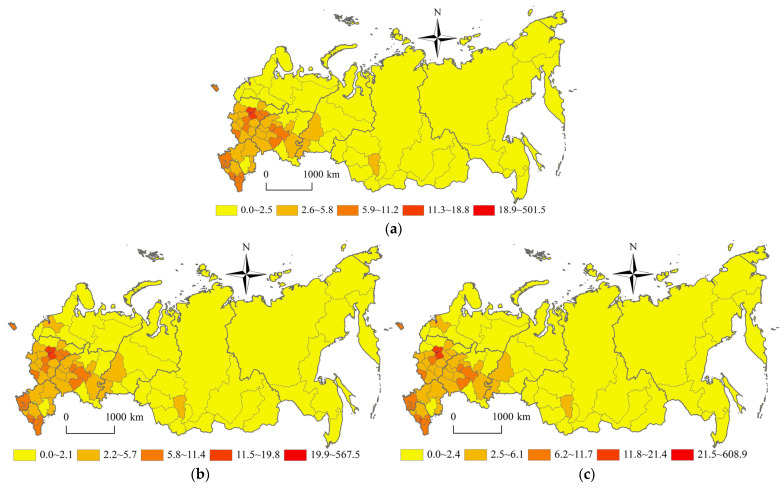
Spatial pattern of Russia’s population concentration degree during 2002–2020. (**a**) 2002 year (**b**) 2010 year (**c**) 2020 year.

**Figure 4:**
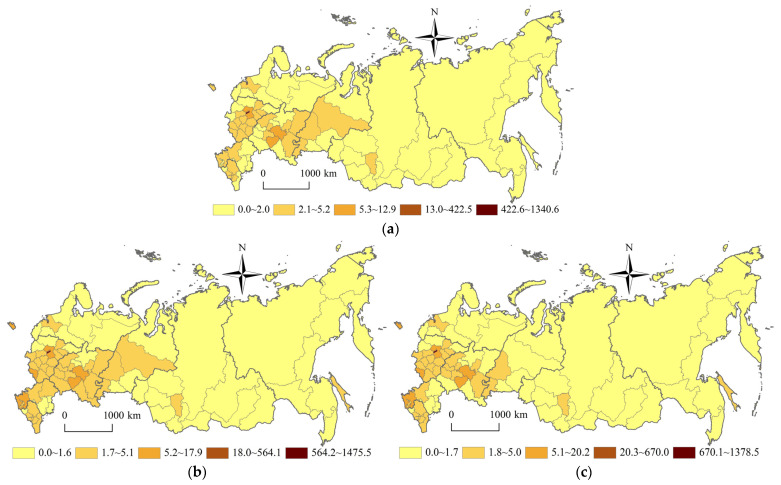
Spatial pattern of Russia’s economic concentration degree during 2002–2020. (**a**) 2002 year (**b**) 2010 year (**c**) 2020 year.

As shown in Figure 3 and Figure 4, Russia’s population concentration degree pattern and Russia’s economic concentration degree pattern have not changed significantly since the 21st century. But they both show an unbalanced development trend, which could be characterized by the differentiation pattern of “High West, Low East” and “High South, Low North” divided by the Ural Federal District. Affected by the original economic base, rich science education resources, convenient transportation infrastructure, etc. the federal subjects, with high population concentration degree and economic concentration degree, are located in Moscow city, St. Petersburg city, with a population of more than one million. Moscow city, with the highest population concentration degree of 608.9, is 60,890 times that of Chukotka Autonomous Area, with the lowest population concentration degree of 0.01. Moscow city, with the highest economic concentration degree of 1475.5, is 73,775 times that of Chukotka Autonomous Area, with the lowest economic concentration degree of 0.02.

The federal subjects, with long-time development, rich energy resources, traditional farming areas, and southern coastal areas, have a high population concentration degree and a high economic concentration degree. The federal subjects, which are located in the polar northern areas, polar forest areas, desert and semi-desert areas, undeveloped energy resources areas, and farming pastoral areas, have low population concentration degrees and low economic concentration degree. Taking the Ural Federal District as the boundary, the high-value areas of population concentration degree and economic concentration degree are distributed in the federal districts of Russian western European areas. In 2020, the population density of Central Federal District, North-Caucasian Federal District, South Federal District and Volga Federal District is 60.4 persons/km^2^, 58.5 persons/km^2^, 36.8 persons/km^2,^ and 28.0 persons/km^2^ respectively. The GDP of Central Federal District, Volga Federal District, Ural Federal District, and North West Federal District is 32.9 trillion rubles, 14.1 trillion rubles, 13.2 trillion rubles, and 10.5 trillion rubles respectively. The low-value areas of population concentration degree and economic concentration degree are located in the federal districts of Russian eastern Asian areas. In 2020, the population density of Siberian Federal District and Far East Federal District is 3.9 persons/km^2^ and 1.2 persons/km^2^ respectively. The GDP of the Siberian Federal District and Far East Federal District is 9.2 trillion rubles and 6.0 trillion rubles respectively. In the western European areas of Russia, the population concentration degree and economic concentration degree are characterized by the differentiation pattern of “High West, Low East” and “High South, Low North”. The high-value areas of population concentration degree and economic concentration degree are distributed in the inverted “V” shape areas of Moscow Region-Vladimir Region-Nizhny Novgorod Region-Republic of Chuvashia-Republic of Tatarstan-Samara Region and Moscow Region-Tula Region-Lipetsk Region-Belgorod Region-Voronezh Region-Rostov Region-Krasnodar Territory-Republic of Adygea-Stavropol Territory. The inverted “V” shape areas are mainly distributed along the Russia’s M-7 highway, M-29 highway, Moscow-Sochi high-speed rail, Moscow-Kazan high-speed rail, etc. Influenced by the space-time convergence effect and the population-economic siphon effect, the spatial polarization effect of the inverted “V” shape axis belt has constantly been enhanced. The population concentration degree and economic concentration degree show the “core-edge” pattern of inverted “V” shape core axial belt areas and non-inverted “V” shape edge areas in the western European areas of Russia. In the eastern Asian areas of Russia, the population concentration degree and economic concentration degree are also characterized by the differentiation pattern of “High West, Low East” and “High South, Low North”. The high-value areas of population concentration degree and economic concentration degree are distributed in the axial belt areas of Omsk Region-Novosibirsk Region-Altay Territory-Kemerovo Region-Irkutsk Region-Republic of Buryatia-Zabaikalsk Territory-Amur Region-Khabarovsk Territory-Primorsky Territory. The axial belt areas are mainly distributed along the Siberian Railway. The low-value areas of population concentration degree and economic concentration degree are located in the northern areas of the Siberian Railway, including the Republic of Sakha (Yakutia), Chukotka Autonomous Area, Magadan Region, Kamchatka Territory, etc. The population concentration degree and economic concentration degree show the “core-edge” pattern with the axis belt of the Siberian Railway as the core and the northern areas of the Siberian Railway as the edge in the eastern Asian areas of Russia.

### 3.3. Spatial Pattern of Population-Economy Inconsistency

**Figure 5:**
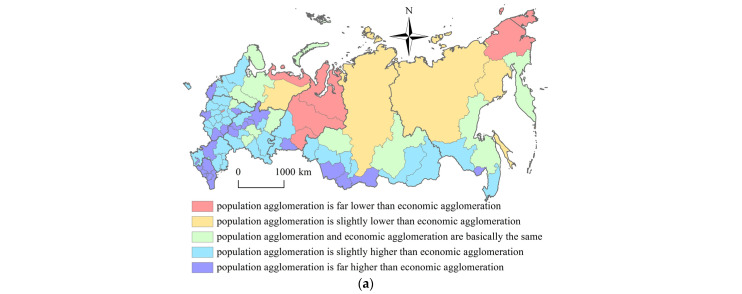

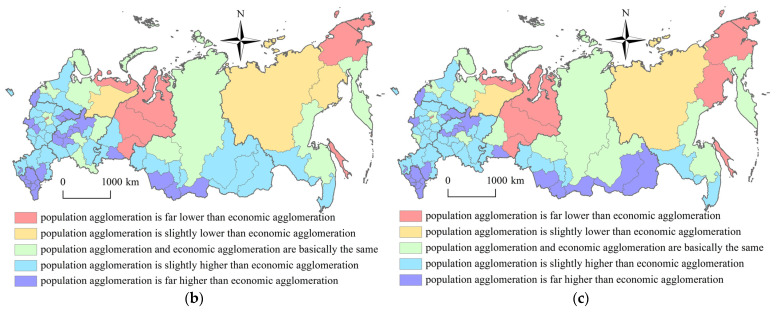
Spatial pattern of population-economy inconsistency in Russia during 2002–2020. (**a**) 2002 year (**b**) 2010 year (**c**) 2020 year.

Combined with the population-economy inconsistency index, we study the consistency and inconsistency of the population distribution and economic development in Russian eighty-three federal subjects. In 2020, the proportion of the federal subjects whose population agglomeration is stronger than economic agglomeration, the population agglomeration and economic agglomeration coordination, population agglomeration is weaker than economic agglomeration, is 72.3%, 14.5%, and 13.2% respectively. Compared with the economic agglomeration, nearly 3/4 of federal subjects have stronger population agglomeration (Figure 5). On these bases, the population-economy inconsistency of the federal subjects could be further divided into nine classes: population and economic coordination type, population backward type, population lagging type, population leading type, population polarization type, economic backward type, economic lagging type, economic leading type, economic polarization type (Table 1).

#### 3.3.1. Classification of Population-Economy Inconsistency Types

As shown in Table 1, Population agglomeration is far lower than economic agglomeration: Moscow city is the economic center of Russia. Tumen Region, Khanty-Mansiysky Autonomous Area, and Yamalo-Nenetsky Autonomous Area are rich in oil and gas resources. The economic indicators values of these federal subjects are much higher than those of other federal subjects. These federal subjects belong to the economic polarization type. Nenetsky Autonomous Area, Chukotka Autonomous Area, etc. are located in the northern areas of Russia. Due to the harsh climate, outdated transportation facilities, etc., the population number of these federal subjects is less than 50,000, and the population density is less than 0.3 person/km^2^. These federal subjects belong to the population backward type. Population agglomeration is slightly lower than economic agglomeration. St. Petersburg city is the second largest city in Russia. It is also a huge comprehensive industrial city in Russia. It belongs to the economic leading type. Magadan Region, Republic of Sakha (Yakutia), etc., have difficulties in resource energy development and social facilities construction. Their population density is less than 0.4 person/km^2^. These federal subjects belong to the population lagging type. Population agglomeration and economic agglomeration are basically the same. Moscow Region, Leningrad Region, Sverdlovsk Region, Krasnoyarsk Territory, Republic of Tatarstan, etc., which have rich energy resources, or have industrial division system, or rely on strong agricultural and industrial complex benefits, or rely on the economic diffusion effect of capital cities, have strong population and economic development levels. These federal subjects belong to the “real” population and economic coordination type. Kamchatka Territory, etc., suffers from the serious population loss, low economic development level, and low industrial structure degree. These federal subjects belong to the “false” population and economic coordination type. Population agglomeration is slightly higher than economic agglomeration. Rostov Region, Republic of Bashkortostan, Chelyabinsk Region, Nizhny Novgorod Region, Samara Region, Novosibirsk Region, Perm Territory, etc., which have pleasant coastal location advantage, or have highly developed agriculture and industry, or become the important multi-dimensional transportation hubs, or have strong scientific and technological innovation potential, have the significant population agglomeration effect. These federal subjects belong to the population-leading type. Jewish Autonomous Area, Republic of Khakasia, Orel Region, Republic of Karelia, etc., have labor shortages, investment lacking, stagnant urbanization, and low social service. The economic indicators values of these federal subjects are much lower than those of other federal subjects. These federal subjects belong to the economic lagging type. Population agglomeration is far higher than economic agglomeration. Although the federal subjects in the northern North-Caucasian Federal District, such as the Republic of Ingushetia, Chechen Republic, Republic of Northern Osetia-Alania, and Republic of Kabardino-Balkaria, have large population density and high geographic concentration. These federal subjects have unstable social status, unstable political situation, frequent ethnic and religious conflicts, low employment status, low education level, low medical facilities, low housing security, and excessive dependence on the central financial transfer payment. The federal subjects, which are adjacent to Mongolia countries, such as Zabaikalsk Territory, Republic of Tyva, Republic of Buryatia, and Republic of Altay, have unbalanced industries proportion, poor transportation infrastructure, unsatisfactory investment environment, and weak international labor division cooperation. These federal subjects all belong to the economic backward type.

#### 3.3.2. Spatial Comparison of Population-Economy Inconsistency

As shown in Figure 5, similar to the spatial pattern of Russia’s population concentration degree and Russia’s economic concentration degree, the spatial pattern of Russia’s population-economy inconsistency has not changed significantly since the 21st century. However, the spatial inconsistency of population distribution and economic development is characterized by a significant differentiation pattern. In the southwest areas of Russia, there are many federal subjects whose population concentration degree is higher than the economic concentration degree. In the northeast areas of Russia, there are many federal subjects whose population concentration degree is lower than the economic concentration degree. In 2020, the federal subjects, which are located in the southwest areas of Russia, have 27.6% of Russia’s land area, 85.7% of Russia’s population, and 85.5% of Russia’s employees, with an average population density of 181.2 persons/km^2^. These federal subjects have 79.9% of Russia’s GDP, 75.1% of Russia’s economic fixed assets, 77.8% of Russia’s industrial output value, 78.7% of Russia’s finance economic balance, and 72.9% of Russia’s fixed capital investment. The population concentration degree of these federal subjects is much higher than that of the economic concentration degree. In 2020, the federal subjects, which are located in the northeast areas of Russia, have 72.4% of Russia’s land area, 14.3% of Russia’s population, and 14.5% of Russia’s employees, with an average population density of 2.9 persons/km^2^. These federal subjects have 20.1% of Russia’s GDP, 24.9% of Russia’s economic fixed assets, 22.2% of Russia’s industrial output value, 21.3% of Russia’s finance economic balance, and 27.1% of Russia’s fixed capital investment. The population concentration degree of these federal subjects is much lower than that of the economic concentration degree. In recent years, the Russian government has introduced a series of policies such as the (Conception on the Regulation of the Migration Process of the Russian Federation) and the (Conception on the National Migration Policy of the Russian Federation before 2025). At the same time, compared with other federal subjects, Moscow city and St. Petersburg city have the highest population number and highest labor resources quality. South Federal District and North-Caucasian Federal District, which are adjacent to the Black Sea and Caspian Sea, have a pleasant coastal climate. Due to the strong attraction effect of these federal districts and federal subjects, the population of northern and eastern areas of Russia flows into the western and central areas of Russia. Among them, the population of the Far East Federal District and Siberian Federal District flow into all the federal districts in the western areas of Russia. The population absorbed by Siberian Federal District from the Far East Federal District could make up for 6% of the lost population in Siberian Federal District. The population of the Ural Federal District flows into the Central Federal District and North West Federal District. The population absorbed by Ural Federal District from the Far East Federal District and Siberian Federal District could make up for 60% of the lost population in Ural Federal District. The population of the North West Federal District flow into the Central Federal District. The population of Volga Federal District flow into the Central Federal District and South Federal District. Therefore, the population concentration degree in the southwest areas of Russia is stronger than the economic concentration degree.

## 4. Discussion

Li found that Russia’s population is concentrated in the regions close to Europe, with the spatial pattern of dense population in the west and sparse population in the east. High-density population concentration areas are distributed in the Volga River coastal area and the southern Ural Federal District, while low-density population sparse areas are located in the western Siberian Federal District, the North West Federal District, the South Federal District and the North-Caucasian Federal District [20]. Feng believed that Russia’s economic development level shows a decreasing trend from the core areas to the edge areas, which presents the developed-sub-developed-developing trend. The core areas are located in the Central Federal District, North West Federal District, Volga Federal District, and Ural Federal District. The sub-core areas are located in the Central Black Soil areas, Volga-Viatka areas, Northern-Caucasus Federal District, and western Siberian Federal District. The edge areas are located in the eastern Siberian Federal District and Far East Federal District [19,34]. In this paper, we select eighty-three federal subjects of the whole territory of Russia. Thus, our research conclusions could not only support Li’s and Feng’s research conclusions and findings, but also are more convincing and comprehensive to a certain extent. In addition, combined with the inconsistency index of the population concentration degree and economic concentration degree, we could classify the population-economic inconsistency types in Russian eighty-three federal subjects. This could have more scientificity, logicality, and focalization compared with analyzing single population distribution analysis or single economic development analysis. This could also get rid of the defects and deficiencies of the single population distribution analysis or single economic development analysis before. However, the data in this paper are from the official publications published by the National Bureau of Statistics of the Russian Federation. The authoritative official statistical yearbooks of Russia are published after 2002. Due to the limitation of data acquisition, this paper could only study Russia’s population pattern evolution trend and Russia’s economic pattern evolution trend during 2002–2020. There are few urban-level indicator data downloaded from the official website of the National Bureau of Statistics of the Russian Federation. Due to the lack of indicator data at the city level, this paper only analyzes Russia’s population pattern and Russia’s economic pattern from the spatial scale of Russian eighty-three federal subjects. It does not study Russia’s population pattern and Russia’s economic pattern at the urban scale. At the same time, although this paper has referred to a large number of books and references, trying to explore the process laws of population distribution and economic development in Russia from the perspective of geography. Due to the problems of population distribution and economic development being too complex, there are still some inaccuracies in grasping scientific problems.

The population concentration degree is greater than that of the economic concentration degree in most of the Russian federal subjects. Russia’s population distribution pattern and Russia’s economic development pattern have presented an unbalanced development trend, which could be characterized by the differentiation pattern of “High West, Low East” and “High South, Low North” divided by the Ural Federal District since the 21st century. Besides, in the southwest areas of Russia, there are many federal subjects whose population concentration degree is higher than the economic concentration degree. In the northeast areas of Russia, there are many federal subjects whose population concentration degree is lower than the economic concentration degree. In the future, Russia should continue to actively promote population and economic coordinated development based on the policies, such as the Vision of the Russian Federation’s National Migration Policy by 2025 and the Strategic Vision of Russia’s Long term Economic and Social Development by 2020. In terms of the population aspect, Russia should promote a rational population flow, which encourages residents to migrate into the Far East Federal District. Russia should also implement a series of population-relevant policies, including eliminating the administrative division barriers, developing the cheap rental market, simplifying the living registration procedures, providing employment information, encouraging temporary employment, realizing internal technology and education migration, improving medical care and other social service systems. With the Republic of Buryatia and Zabaikalsk Territory redistributing from the Siberian Federal District into the Far East Federal District in 2018, the Far East Federal District has huge development potential in Russia. Under the background of economic integration into the Asia Pacific region, Far East Federal District should implement the (Economic and Social Development Strategy for the Far East Federal District and Baikal region before 2025) in the future. Relying on developing key energy endowment, eliminating transportation bottlenecks, eliminating power grid bottlenecks, and eliminating communication bottlenecks, Far East Federal District should do its best to stabilize the local population number. Then Far East Federal District should realize the internal residents migration from the southern areas to the northern areas, and gradually implement unconventional foreign residents’ immigration. In terms of the economic aspect, Russia should accelerate the industrial optimization layout, realizing the multi-polar economic development. Russia’s Science Cities should play the important role in leading industrial technological development. Russia’s resource-shrinking regions should accelerate industrial upgrading and industrial transformation. Russia’s border cities should implement the opening development strategy, carrying out all-around economic and trade cooperation with neighboring countries. Russia’s northern areas should accelerate the construction of the Arctic Channel. Russia’s capital, port cities, and southern caring areas should deepen the tertiary industry development, changing the economic structure dominated by energy exports, and realizing the transformation from a resource-based economy to an innovative economy. The whole of Russia should vigorously strengthen the regional infrastructure construction, especially the ports construction, airports construction, information facilities construction, high-speed rails construction, etc., so as to improve the land space connection efficiency, compactness degree, and integration degree. As for the western European areas of Russia, they should strengthen the construction and development of metropolitan areas and urban agglomerations, in line with the current trend of urbanization development and information technology development of the world. The western European areas of Russia should not only strengthen their own economic advantages, but also attract foreign financial resources. They should focus on the development of three super large urban agglomerations, including the Moscow super large urban agglomeration, St. Petersburg super large urban agglomeration, Yekaterinburg-Chelyabinsk-Ufa-Perm super large urban agglomeration, as well as other large urban agglomerations, including the Kazan-Samara-Togliatti-Ulyanovsk-Izhevsk large urban agglomeration, Rostov on Don-Krasnodar large urban agglomeration, Nizhny Novgorod large urban agglomeration, etc. As for the eastern Asian areas of Russia, combined with the growth pole theory and the point-axis theory, they should focus on cultivating the economic growth poles along the Siberian Railway to form the point-axis system. The eastern Asian areas of Russia should focus on building the Novosibirsk Railway Hub Economic Zone with Novosibirsk as the core, the Baikal Lake Economic Zone with Ulan Ude and Irkutsk as the cores, the Coastal Economic Zone with Vladivostok, Nahodka and Khabarovsk as the cores, so as to continuously narrow the economic development gap with the western European areas of Russia.

## 5. Conclusions

Combined with the panel data of population, GDP, land area, etc., Russia’s population pattern evolution trend, Russia’s economic pattern evolution trend, Russia’s spatial inconsistency types of population distribution and economic development, were studied by using the gravity center analysis, geographic concentration degree, inconsistency index, etc. The conclusions are as follows:

Russia’s geographical gravity center is located in Chelyabinsk Region in the Ural Federal District. Russia’s population gravity center and Russia’s economic gravity center are both located in the northwest direction of Russia’s geographical gravity center. Russia’s population gravity center and Russia’s economic gravity center have been gradually migrating away from Russia’s geographical gravity center. The distance between Russia’s population gravity center and Russia’s geographical gravity center has been increasing from 444 km in 2002 to 494 km in 2020. The distance between Russia’s economic gravity center and Russia’s geographical gravity center has been increasing from 368 km in 2002 to 402 km in 2020. The spatial migrating degree, spatial migrating scope, and spatial migrating distance of Russia’s economic gravity center are larger than those of Russia’s population gravity center. Russia’s economic imbalance degree is greater than Russia’s population imbalance degree. Russia’s economic expansion process is relatively rapid, and Russia’s population flow process is relatively slow. Russia’s population gravity center and Russia’s economic gravity center are migrating towards the northwest direction, which is closely related to Russia’s geographical environment, resource endowment, development intensity, policy system, productivity layout, etc.

Russia’s population concentration degree pattern and Russia’s economic concentration degree pattern have not changed significantly since the 21st century. But they both show an unbalanced development trend, which could be characterized by the differentiation pattern of “High West, Low East” and “High South, Low North” divided by the Ural Federal District. Taking the Ural Federal District as the boundary, the population concentration degree and economic concentration degree of the western European areas of Russia are higher than those of the eastern Asian areas of Russia. In western European areas of Russia, the population concentration degree and economic concentration degree show the “core-edge” pattern of inverted “V” shape core belt areas (Moscow Region-Samara Region and Moscow Region-Stavropol Territory) and non-inverted “V” shape edge areas. In the eastern Asian areas of Russia, the population concentration degree and economic concentration degree show the “core-edge” pattern with the axis belt of the Siberian Railway (Omsk Region-Primorsky Territory section) as the core and the northern areas of the Siberian Railway as the edge.

Compared with economic agglomeration, nearly 3/4 of federal subjects have stronger population agglomeration. Similar to the spatial pattern of Russia’s population concentration degree and Russia’s economic concentration degree, the spatial pattern of Russia’s population-economy inconsistency has not changed significantly since the 21st century. However, the spatial inconsistency of population distribution and economic development is characterized by significant differentiation characteristics. In the southwest areas of Russia, there are many federal subjects whose population concentration degree is higher than the economic concentration degree. In the northeast areas of Russia, there are many federal subjects whose population concentration degree is lower than the economic concentration degree. On these bases, the population-economy inconsistency of the federal subjects could be further divided into nine classes: population and economic coordination type, population backward type, population lagging type, population leading type, population polarization type, economic backward type, economic lagging type, economic leading type, economic polarization type.

## Figures and Tables

**Table 1:** Classification of population-economy inconsistency types in Russian federal subjects.

Category	Types	Meaning	Federal Subjects						
0~0.5	population agglomeration is far lower than economic agglomeration	population backward or economic polarization	Moscow city	Tumen Region	Khanty-Mansiysky Autonomous Area	Yamalo-Nenetsky Autonomous Area	Nenetsky Autonomous Area	Sakhalin Region	Chukotka Autonomous Area
0.5~0.8	population agglomeration is slightly lower than economic agglomeration	population lagging or economic leading	Saint-Petersburg city	Republic of Komi	Magadan Region	Republic of Sakha (Yakutia)			
0.8~1.2	population agglomeration and economic agglomeration are basically the same	population and economic coordination	Moscow Region	Irkutsk Region	Belgorod Region	Republic of Tatarstan	Leningrad Region	Murmansk Region	Tomsk Region
			Sverdlovsk Region	Krasnoyarsk Territory	Arkhangelsk Region	Khabarovsk Territory	Kamchatka Territory		
1.2~2.0	population agglomeration is slightly higher than economic agglomeration	population leading or economic lagging	Penza Region	Samara Region	Orel Region	Kaliningrad Region	Novosibirsk Region	Ryazan Region	Tver Region
			Saratov Region	Kemerovo Region	Voronezh Region	Vladimir Region	Tambov Region	Tula Region	Kaluga Region
			Kursk Region	Orenburg Region	Rostov Region	Astrakhan Region	Perm Territory	Primorsky Territory	Amur Region
			Lipetsk Region	Volgograd Region	Smolensk Region	Novgorod Region	Republic of Khakasia	Omsk Region	Jewish Autonomous Area
			Republic of Bashkortostan	Chelyabinsk Region	Krasnodar Territory	Nizhny Novgorod Region	Republic of Udmurtia	Republic of Karelia	Ulyanovsk Region
			Vologda Region	Yaroslavl Region					
more than 2.0	population agglomeration is far higher than economic agglomeration	population polarization or economic backward	Kirov Region	Bryansk Region	Republic of Altay	Republic of Chuvashia	Kurgan Region	Pskov Region	Republic of Adygea
			Republic of Tyva	Chechen Republic	Kostroma Region	Zabaikalsk Territory	Ivanovo Region	Altay Territory	Republic of Ingushetia
			Stavropol Territory	Republic of Dagestan	Republic of Mordovia	Republic of Buryatia	Republic of Karachaevo-Cherkessia	Republic of Kalmykia	Republic of Kabardino-Balkaria
			Republic of Northern Osetia-Alania	Republic of Marii El					

## Data Availability

The data used to support the findings of this study are available from the corresponding author upon reasonable request.
